# Multilayer subwavelength gratings or sandwiches with periodic structure shape light reflection in the *tapetum lucidum* of taxonomically diverse vertebrate animals

**DOI:** 10.1002/jbio.202200002

**Published:** 2022-03-20

**Authors:** Lidia Zueva, Astrid Zayas-Santiago, Legier Rojas, Priscila Sanabria, Janaina Alves, Vassiliy Tsytsarev, Mikhail Inyushin

**Affiliations:** 1 Universidad Central del Caribe School of Medicine, Bayamon, Puerto Rico, USA; 2 University of Maryland School of Medicine, Baltimore, Maryland, USA

**Keywords:** eye, subwavelength gratings, tapetum lucidum, vision

## Abstract

Eye shine in the dark has attracted many researchers to the field of eye optics, but the initial studies of subwavelength arrangements in tapetum began only with the development of electronic microscopy at the end of the 20th century. As a result of a number of studies, it was shown that the reflective properties of the tapetum are due to their specialized cellular subwavelength microstructure (photonic crystals). These properties, together with the mutual orientation of the crystals, lead to a significant increase in reflection, which, in turn, enhances the sensitivity of the eye. In addition, research confirmed that optical mechanisms of reflection in the tapetum are very similar even for widely separated species. Due to progress in the field of nano-optics, researchers now have a better understanding of the main principles of this phenomenon. In this review, we summarize electron microscopic and functional studies of tapetal structures in the main vertebrate classes. This allows data on the microstructure of the tapetum to be used to improve our understanding of the visual system.

## Graphical Abstract

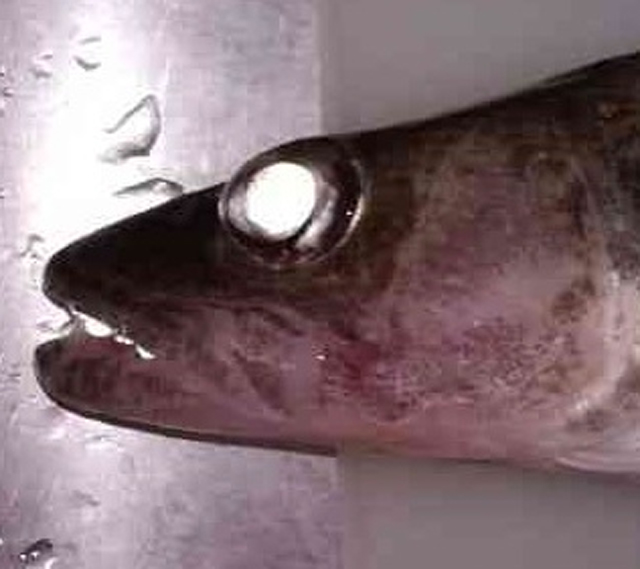

The reflective properties of the tapetum are due to their specialized cellular subwavelength microstructure (photonic crystals). Optical mechanisms of reflection in the tapetum are very similar even for widely separated species. In this review we summarize electron microscopic and functional studies of tapetal structures in the main vertebrate classes. This allows data on the microstructure of the tapetum to be used to improve our understanding of the visual system.

## 1 | INTRODUCTION

**FIGURE 1:**
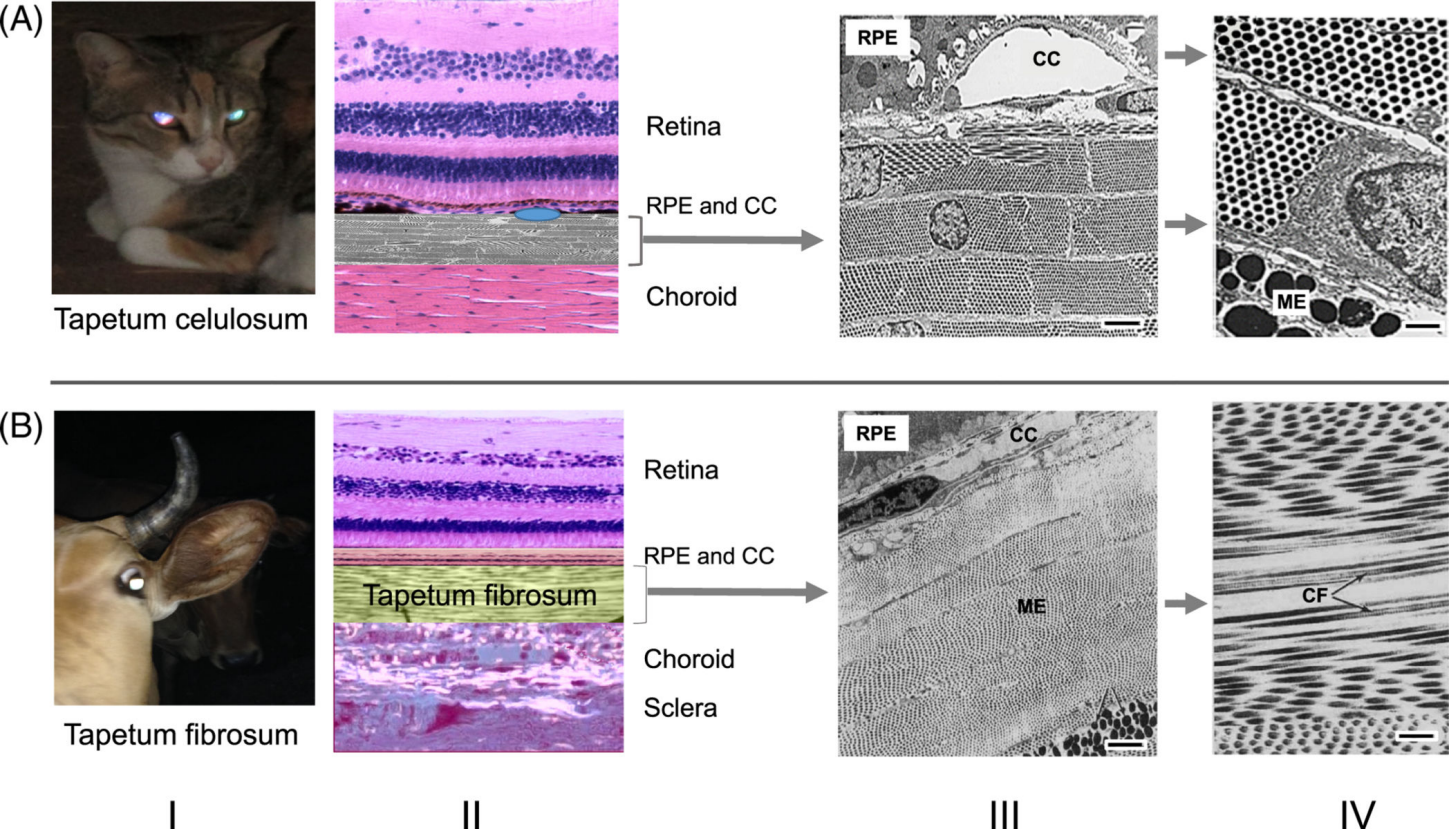
Choroidal tapetums. (A) The *tapetum lucidum cellulosum* of carnivores, seals, and lower primates is made of specialized melanocytes, shown here at different amplifications (III and VI). In these animals, high amplification reveals highly organized gratings (see text) composed of rodlets formed inside the tapetum cells (cat structures are shown as an example). (B) The *tapetum fibrosum* of ungulates, shown at different amplifications (III and VI). In these animals, melanocytes produce extracellular protein fibers that form long stacks with a highly organized hexagonal structure in cross-section (see text). I, an example of light reflection from the eye; II, retinal layer scheme showing the position of the tapetum; III, electron microscopy of a tapetum layer (×1000); IV, electron microscopy of tapetum layer (×5000). A-III, -IV, modified from Braekevelt, 1990 (with permission) [4]; B-III, -IV, modified from Braekevelt, 1986 (with permission) [5]. ME, melanin granules; CC, choriocapillaris; RPE, retinal pigment epithelium; CF, filaments. Bars: A-III, 500 nM; A-IV, 200 nm; B-III, 500 nm; B-IV, 200 nm

The *tapetum lucidum* is a biological reflector system that is a common feature in the eyes of many vertebrates, although some species (mostly diurnal) lack this structure [1, 2]. The reflection of light that has already passed through the retina provides the light-sensitive retinal cells with a second chance for photoreceptor stimulation, thereby enhancing visual sensitivity at low light levels, which is especially important for nocturnal animals. In vertebrates, the *tapetum lucidum* exhibits a diverse structure, organization, and composition. Its location may be choroidal, that is, inside the vascular layer of the eye (choroid), lying between the retina and the sclera (choroidal tapetum type) or directly in the retina around the photoreceptors (retinal-type tapetum). While there are different types of choroidal tapetum, *tapetum cellulosum* (composed of a specialized cell layer) occurs only in certain mammals, including carnivores, seals, certain lower primates [1, 2], and possibly in certain teleost fish [3]. The reflections are generated by specialized reflective structures made of organic material. The nature of the reflective material varies among species, and their descriptions are sometimes contradictory. In *tapetum cellulosum*, the reflective material, usually in the form of rodlets (needles) of a special shape, is contained inside the cells of the choroid. Cells in this tissue are usually flat and stacked on top of each other, with their faces parallel to the plane of the retina [1] (Figure 1A). In the ungulate *tapetum fibrosum*, the reflective material is a fibrous substance also forming stacks in the space between and outside the tapetum cells (Figure 1B). In birds (Figure 3A), reptiles, amphibians, and fish (Figure 3B) the retinal-type tapetum also consists of specialized highly organized nanostructures inside specialized cells localized around photoreceptors as an integral part of the retinal layer. Besides the tapetum, in some fishes and amphibians, additional mirrors (argentea) are formed in the sclera and, to some extent, in the iris. Histologically, the argentea and the choroidal tapetum are situated in opposite (scleral versus vitreal) parts of the choroid, respectively. It is understood that special organic mirrors made of periodic lipid-, protein-, or guanine-based nanostructural materials [2, 6–8] reflect light in the tapetum and argentea of diverse species. In this review, we focused on the similarities and differences in tapetal reflecting nanostructures found in different vertebrate species using electron microscopy and on the possible mechanisms of light reflection from such structures that have been described in the literature.

## 2 | RODLETS IN *TAPETUM LUCIDUM CELLULOSUM*

**FIGURE 2:**
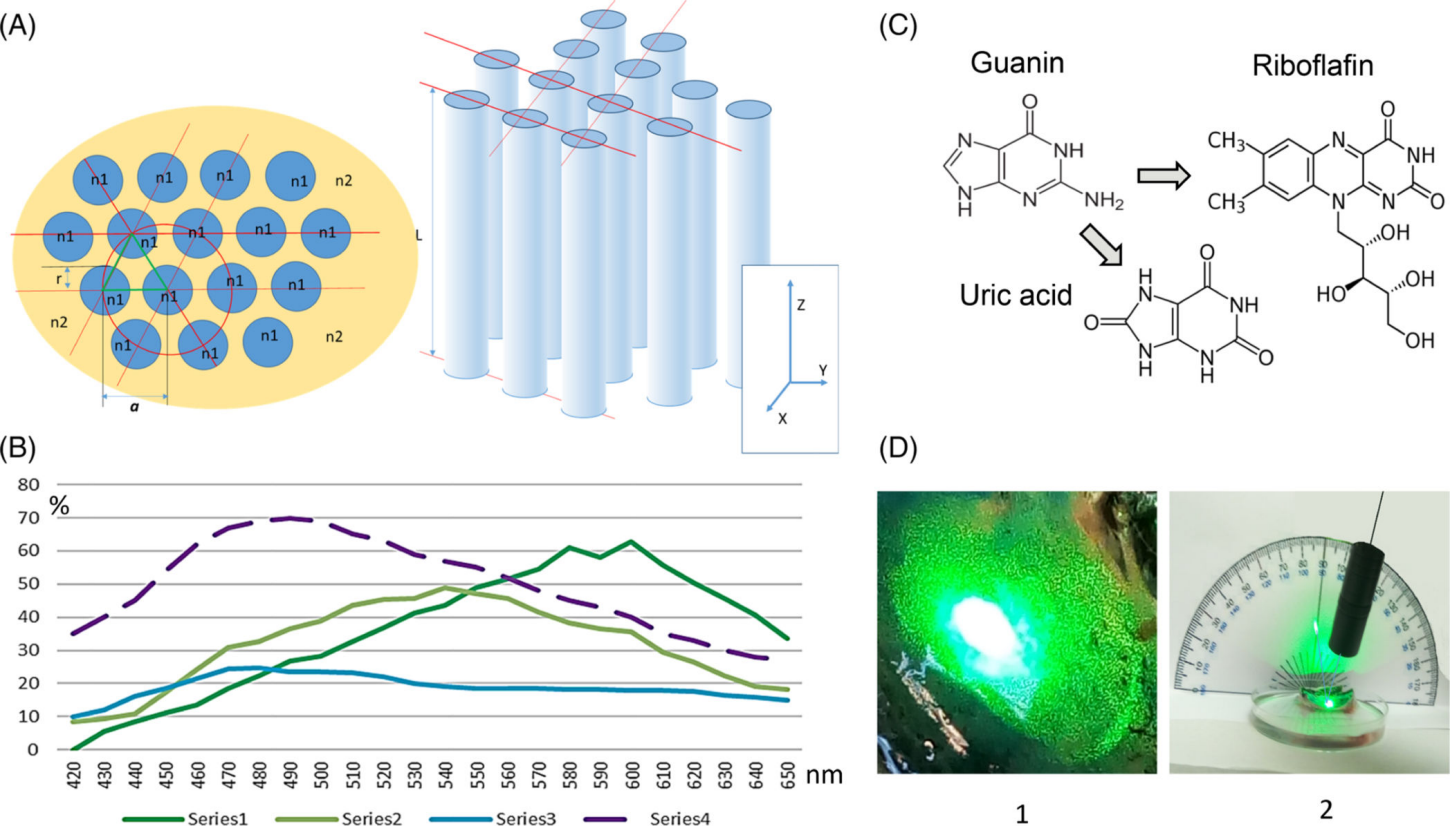
(A) Rodlet (fiber) organization in the tapetum of ungulates. Parallel rodlets are precisely organized in bundles, with all bundles lying parallel to the plane of the choroid (Z-coordinate). Nearby rodlets form a periodic hexagonal structure (grating) in X-Y plane, which is clearly subwavelength for visible light, as the distance between each rodlet is ~100 nm, with the diameter of the rodlet ~100 nm. (B) Reflection of white light from isolated cat tapetum (in %), based on: (Gunter et al [9]; bluish series 3) and (Weale [10]; yellowish series 2, greenish series 1), with modifications. Rod pigment from cat eye sensitivity curve [17] for reference. (C) Riboflavin is the rodlet material in carnivores and is based on the guanine molecule [18], as well as uric acid, which is present in the tapetum of some fish. D, left. Visible reflecting fibers in cow tapetum (ungulates) lit with a green laser. D, right. Anomalous diffuse reflection from bovine tapetum (see text)

There have been many studies of this tapetum type. The spectral reflectivity of cat tapetum was thoroughly studied, because of the general interest of cats to humans and their familiarity as research subjects [4, 9–16]. The *tapetum lucidum* in cats is thought to be composed of specialized melanocytes that are differentiated from standard choroidal melanocytes [15]. This structure consists of 15 to 30 layers of flat cells in the center of the tapetum, while on the periphery the thickness attenuates to only 1 to 2 layers. These flat cells, rectangular in shape in vertical cross-section and 3 to 4 μm thick, are filled with high-electron-density rodlets clearly visible under an electron microscope [4, 12]. Parallel rodlets (Figure 1A–III and -IV; Figure 2A) appear to be precisely organized in bundles, each rodlet ~5 μm in length, all bundles lying parallel to the plane of the choroid, and the angle between each bundle seemingly arbitrary [4, 12, 19]. Interestingly, the famous histologist Max Schultze [20], using only a light microscope, described small rodlets organized in bundles and the reflections from these bundles due to interference. Each rodlet in the bundle is precisely arranged in a hexagonal (60°) lattice pattern (Figure 1A–II and -IV; Figure 2A), with a uniform spacing between adjacent rodlets. The rodlets themselves have a constant diameter, but researchers employing electron microscopy report diameters of 190 to 230, 100 to 120, or 100 nm, as well as mean distances between rodlet centers of 400 or 150 nm [4, 12, 19].

These inconsistencies may be due to variability from specimen to specimen or may be an artifact of the method. It should be noted that Pedler and Braekevelt used Araldite (epoxy) medium for slice embedding, while Bernstein and Pease used old *n*-butyl methacrylate; however, their results were similar [4] [19], suggesting that embedding artifacts are not responsible for the inconsistencies between reports. Similarly, the fine structure of the canine *tapetum lucidum* was also studied, and it was found to be very similar to the rod gratings discovered in cats [21], with subwavelength (for visual light) distances between elements of the gratings, similar to the distances described in cats. Analogous structures have also been described in ferrets [22, 23].

## 3 | RODLET MATERIALS IN CARNIVORES

Riboflavin was found in lemur eyes [24], as well as in cat eyes. Cat choroid cells have a high level of riboflavin (vitamin B2), 71 mg/g fresh tissue, which is higher than the level in liver, 26 mg/g [25]. Another flavin (7a-hydroxyriboflavin) is also present in cat choroid cells [16]. During development, at the third postnatal week, cat choroid melanocytes are converted to tapetal cells, and at this time the tapetal cells change their fluorescence from green DOPA fluorescence (formaldehyde-induced) to orange-yellow autofluorescence, consistent with the fluorescence of authentic flavins [15]. It is possible that these rodlets are composed mainly of the crystalline form of flavins (B2 and 7a-hydroxyriboflavin, Figure 2C), and it was found that riboflavin and rodlet material have a similar refractive index (R.I.). The crystalline form of riboflavin has a refractive index parallel to the crystal long axis of ~1.538 and perpendicular to the long axis of 1.733, [14, 26] determined the refractive index of cat tapetum rodlets. The rodlets were first separated by gently homogenizing pieces of tapetum in water with subsequent centrifugation, and their refractive index was found to be ~1.69, while the tapetum cell cytoplasm R.I was estimated as 1.33 [14]. It was also found that each rodlet in cats is surrounded by its own membrane, which is rich in zinc (Zn), which in turn is associated with taurine [27] or cysteine [28], both known as physiological membrane stabilizers. Similar materials were described in the rodlet membranes of dogs and ferrets [23]. In vertebrates, riboflavin is synthetized mainly from guanine [18]. This is interesting, because light reflectance in many other vertebrate species is also based on guanine or its products, as guanine is a natural biological material with one of the highest known refractive indices, 1.89 [7].

## 4 | COLLAGEN FIBER BUNDLES IN TAPETUM FIBROSUM

This tapetum type is formed as a special extracellular layer (Figure 1, II). The reflective tissue in this tapetum type consists of lamellae (arrays and bundles) of extracellular fibrils running parallel to the retinal surface and situated under the retinal pigment epithelium (RPE) cell layer [1, 2, 29, 30]. In horse, the reflective lamellae are spread over almost the entire ocular fundus and are thicker in the horizontal band dorsal to the disc. This band is covered by transparent unpigmented RPE, allowing reflection, which suggests that it is responsible for both mesopic and scotopic vision. This structure consists of multiple parallel layers of (putative) collagen fibrils [30]. Sheep tapetum consists of several hundred of these layers built of flexible fibers ~150 nm in diameter and running predominantly horizontally over most of the tapetum.

These layers are probably made of collagen (as they display standard cross striations of collagen, and hydroxyproline makes up ~60% of the dry weight of the tissue) [31]. Similarly, bovine tapetum fibrils display the normal cross striations characteristic of native collagen. According to another study, bovine fibrils (Figure 1B) are 200 nm in diameter and arranged in a hexagonal pattern (similar to Figure 2A), with a center-to-center spacing of also ~200 nm; the diameter and spacing of these fibrils allow the reflection of interfering light [29]. From the diameter and interfibrous distance of the collagen fibrils, the wavelength of the light reflected from the horizontal band of the tapetum was estimated to be ~468 nm. Therefore, the light reflected from the tapetum should be more effectively absorbed by rods than by cones and should not interfere with photopic vision (similar numbers were found for cats, Figure 2B). We performed simple experiments studying the reflection from bovine tapetum in vitro. Figure 2D (left) shows a green laser light beam (532 nm, 1.2 mm in diameter) pointing at the bovine tapetum in vitro, while the light from this point spreads to nearby bundles, making them visible. Also, in Figure 2D (right) there is a diffuse reflection from the tapetum, and the angle of this reflection (the center of the reflective spot) does not coincide with the angle of mostly specular reflection from the tapetum-water interface (the retina was removed mechanically), suggesting that this may be a case of anomalous reflection not predicted by Snell’s law [32]. However, a broader range of reflectance experiments was performed in cats.

## 5 | REFLECTANCE FROM CAT TAPETUM

### 5.1 | Spectrum of reflectance

With white incident light, different researchers described the spectrum of the reflected light differently. Measured in situ in cat eye, one group determined the reflectance to be “yellowish green” [33], while another [34] termed it “green,” and the reflectance is known to vary considerably from cat to cat in the yellow-green spectral area. Working with extracted tapetum mounted in glycine, Gunter et al. [9] found that the reflection appears bluish, because it reflects more effectively in the blue-green diapason, while the reflectance maximum was at ~470 nm (Figure 2B). These authors found that some reflectance exists also in the infrared diapason. Weale [10], working both with isolated tapetum and with animal eyes in situ, found that, for the first half hour after extraction, the reflectance from tapetum is yellow-green but then slowly changes to blue. This bluish reflection could be once again returned to yellow-green if the tapetum cells are irrigated with saline, and it was suggested that the effect was due to the dehydration of the cells in isolated tapetum [10]. In situ the reflectance always seemed to be yellow-green. Weale also found that the spectrum of reflected light is characteristic for specific adult cats but generally may be divided into two groups with relatively similar mean values, which can be characterized as “greenish” or “yellowish” eye reflection (Figure 2) [10]. Yellowish reflectance appears to be more effective and reaches a maximum at 580 nm, while greenish has a maximum yield at 450 nm. Fourteen-day-old kittens were found to be without reflecting tapetum [10]. Results from Gunter et al [9, 10] are shown in Figure 2B as graphs with greenish, yellowish, and blue colors. The graph on the sensitivity of the rod pigment rhodopsin, which defines scotopic vision [17], is also shown (Figure 2B, violet slash line) as a reference. An interesting illustration of the variety of cat eye reflection spectra can be found online (https://qph.fs.quoracdn.net/mainqimg-14b29f70240b976094b235d8df32149f-lq; accessed on December 15, 2021). It is also known that many white (albino) and Siamese cats have no functional tapetum, with no bright green-yellow reflection from the tapetal zone, nor is there any choroidal pigmentation. Observed in vivo, the tapetal zone in these cats has the appearance of a jungle of red blood vessels, and the reflectance appears orange-red and is not very efficient [35].

Interestingly, in certain other species, the spectrum of the reflection from the eyes in vivo may change with the season. In summer, the reflection from the eyes of arctic reindeer is yellowish, whereas in winter it is deep blue [36]. This effect was attributed to mechanical changes to the reindeer tapetum cells due to increased intra-ocular pressure in winter, probably produced by permanent pupil dilation (because of low light levels in winter), which blocks ocular drainage. Mechanical stretching of tapetum can reproduce this effect [36]. The stretchingrelated changes in the reflected light spectrum in reindeer are similar to what was found in isolated cat tapetum [10] and may be attributed to spacing changes between rodlets. Coles [14], who had studied isolated cat tapetum and was not interested in the overall reflectance spectrum, found that red light was mainly reflected from deep layers of the tapetum, while blue light was reflected from the upper cell layer near the vitreal surface. He suggested that this effect is due to the differences in rodlet spacing between lower and upper layers, as the distance between rodlets is about 20% greater in the lower layers of the tapetum [4, 12, 19].

### 5.2 | Effectiveness and angle of reflection from cat tapetum

Previous studies of cat tapetum reflection and its effectiveness were made on the assumption that the tapetum is a diffusing (scattering) reflector but not a specular mirror. However, unlike diffusing reflectors, cat tapetums retain their polarization in reflected light, representing a clear anomaly from a classical optics point of view [37, 38]. In any case, until 20 years ago, there was insufficient knowledge about surfaces with specially designed “anomalous” reflection(s), and even retroreflection from cube-corner microprismatics or from spherical retroreflectors, which are now commonly used in signaling for traffic control, were not well known. A majority of authors agreed at that time that the tapetum within the cat fundus behaves essentially as an ideal diffuser [10, 14, 37, 38], and this assumption was made because of the so-called “defocusing” test (Bonds, 1974). This assumption probably affected experiments that gave contradictory or inexplicable results.

Gunter et al [9] illuminated tapetum using the lens and light source from a spectrophotometer and found that the reflectance was weak, with maximum reflection of the incident light in the range 15% to 24%, which was much less than the reflection from the empty cover glass. With that quality of reflection, Gunter et al [9] suggested that it can be assumed that the tapetum reduces to some degree the absolute threshold of vision. But in their other work with in vivo measurements of the visual threshold in cats, they found that, on the contrary, reflection added to the absolute threshold level. Weale [10] showed in in situ cat eyes that the reflection of white light from the cat’s isolated “greenish” tapetum (at normal incidence to the tapetum) near the center was ~65% of the reflection from the white diffuse surface (represented by dry magnesium oxide powder), while the reflection at other angles was significantly less. The reflectance was found to be at a maximum in the tapetum center and to diminish near the border.

Studies with cats in vivo have found that reflection was “considerable” or “very efficient,” but no quantitative study was made in these cases [33].

Coles [14] used a narrow collimated “pencil” of light to study the reflectance of isolated cat tapetum and found that it is composed of multiple reflecting domains (<6 μM in diameter each) that seemed to correspond to the separate intracellular rodlet bundles seen in electron micrographs (Figure 1A). Each domain reflected light back to the light source when the incidence was within the angle of 15 to 20° from vertical, and this was explained by the domain having a set of “reflecting planes” [14]. While some of the domains had reflecting planes, even at 36° inclination, the domains illuminated at about 22° of incidence sometimes gave two reflections of roughly equal brightness—one of them back to the source and the other symmetrically from the source at an angle equal to the incidence angle, thus contradicting Snell’s law. There was no satisfactory explanation offered for this phenomenon. The author interpreted this strange effect as reflectance from “horizontal” and “vertical” planes of the same domain mirror [14]. In the same publication, Coles also found that red light was mainly reflected from the deep layers of the tapetum, while blue light was reflected from the upper cell layer lying near the vitreal surface, and this effect was attributed to differences in the rodlet structural spacing between deeper and shallower layers.

## 6 | RETINAL-TYPE TAPETUM (BIRDS, REPTILES, AMPHIBIANS AND FISH)

**FIGURE 3:**
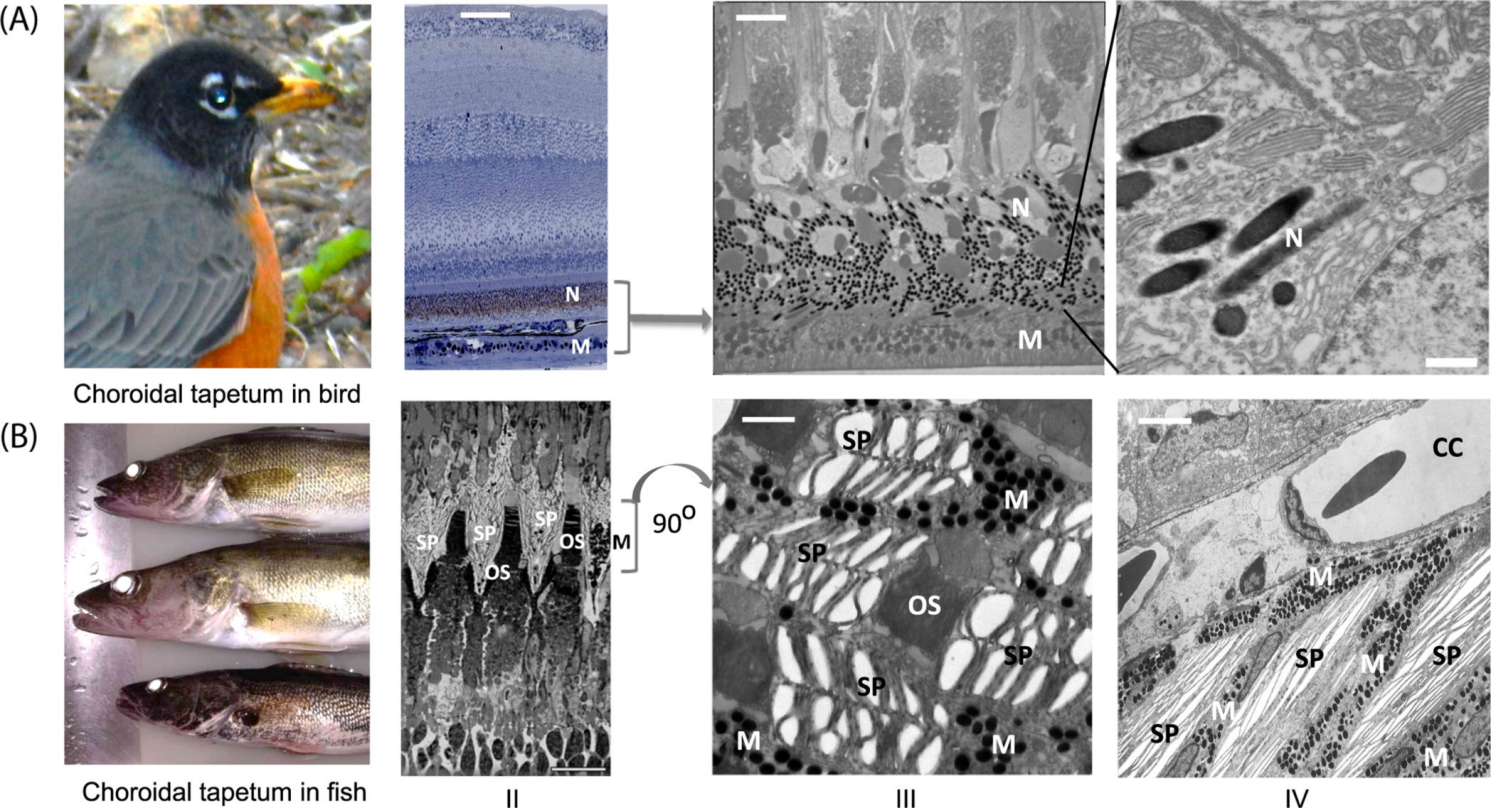
Retinal-type tapetum. (A) Retinal bird *tapetum lucidum* is made of specialized melanocytes, shown here at different magnifications (II, III, and IV). In these animals, high magnification shows spatially well-organized needles (see text) in the cell bodies and apical processes of the melanocytes surrounding the photoreceptors. I, an example of light reflection from bird eye. Pied flycatcher structures: II, retinal layer scheme showing the position of the tapetum in retina; III, electron microscopy of tapetum (×1000); IV, electron microscopy of tapetum (×5000). (B) Retinal tapetum with reflective crystallites (sets of plates) in fish, shown at different amplifications (II, III, and IV). II, overall structure of retinal tapetum in anchovy (low magnification); III, crystallites in shark (5000x); IV, crystallites around the outer segment of a photoreceptor in anchovy (×5000) A-II, -III, and -IV, original images from the authors; B-III, -IV, modified from Kondrashev et al [55] (with permission; B-III, modified from Braekevelt et al [43] (with permission). CC, choriocapillaris; M, melanin granules; N, needles; OS, outer segment of photoreceptor; RPE, retinal pigment epithelium; SP, stack of plates (crystallite). Bars: A-II, 100 μm; A-III, 4 μM; A-IV, 500 nm; B-II, 10 μm; B-III, 500 nm; B-IV, 4 μm

Retinal-type tapetum is also formed by pigment epithelial (RPE) cells forming a layer in the choroid but with long apical processes of these cells extending to form a casing around the photoreceptor outer segments (OSs) in the visual layer of the retina. The visual cell layer contains cones (or double cones) and/or rods, and their OSs are surrounded by the apical processes of the pigment epithelium cells with reflective elements. RPE cell bodies contain both melanin granules (melanosomes) and reflective elements (Figure 3A–III and –IV). Thus, this tapetum type provides not only reflectance back to the photoreceptors but also optical insulation of receptor OSs from each other with reflective material in the RPE apical processes. In some deep-water teleost fish with only rodcontaining (cone-less) retina, RPE cells have short apical processes, and their tapetums resemble a simple (single-layer) choroidal tapetum [39]. Melanosomes, while found throughout the epithelial layer, are scarce centrally and more numerous peripherally (Figure 3A–III). The RPE cells are usually columnar cells tall enough to have a large accumulation of reflective material within (sometimes very tall, as in goldeye [*Hiodon alosides*] and walleye [40, 41]. The amount of reflective material in an apical process change with luminance, due to the necessity for this tapetum type to accommodate the photoreceptor OSs during retinomotor movements [42]. Specialized nanostructures that were suggested as reflective elements are found in RPE cells for this tapetum type. For the retinal tapetum in different animals, three categories of reflective elements are recognized in the literature: (1) spheres, (2) needles (spindles), (3) crystallites (bundles of plates) [2, 4, 43–45]. Highly dissimilar substances have been reported as forming these nanostructures: guanine, uric acid, lipid (glyceryl tridocosahexaenoate), pteridine (7,8-dihydroxanthopterin), and melanoid (a tetramer of 5,6-dihydroxyindole-2-carboxylic acid combined with decarboxylated S-adenosylmethionine) [46–51]. These findings have also been reviewed [3, 7, 8].

### 6.1 | Spheres

In RPE cells, each sphere is usually 300 to 500 nm in diameter (about the wavelength of visible light) and situated close to each other, sometimes touching one another and forming a symmetric hexagonal structure (similar to rodlet planes, Figure 2A). The boundary layer of spheres contains a membrane made of a dissimilar material that is slightly thicker than ordinary cytomembrane. In sea trout, chromatography suggests that the main content of these spheres is triglyceride-C22, which is similar to tridocosahexaenoin, with a reflective index near 1.49, and it was concluded that reflection from spheres is due to a Mie scattering effect (Arnott et al [46]). Similar spheres have been described in many fish species [40, 51, 52] and even in birds [44], while there is no other confirmation of birds having this type of reflective element (and electronic microphotographs from this latter work are not very convincing).

*Needles* are prevalent as reflective elements in the RPE cells of birds, reptiles, and amphibians. The eye reflection from American robin (Figure 3A–I) illustrates the reflection from bird tapetum. As a beautiful example, microphotographs of the retinal layers of the pied flycatcher bird with reflective needles in RPE cells is presented in (Figure 3A–II, -III, IV). At low magnification, the visible light microphotograph shows that reflective elements (brown) are presented in the apical processes of the RPE between the OSs of cones and in RPE cell bodies centrally, while melanin granules (black) are seen in the RPE only distally (Figure 3A–II). The same distribution is present in an electron micrograph of the tapetum (the needles look blacker, while the melanin granules look less electron dense). The needles in apical processes can be seen as long shafts, while in the RPE the cell bodies look like spheres, as all needles lie parallel to the visual plane (Figure 3A–III). Thus, the majority of needle shafts in the RPE cell body are located parallel to the visual plane (with spherical sections), while the apical processes of RPE needles are located perpendicular to the visual plane, serving as reflective separators between photoreceptor OSs (Figure 3A–III and -IV). The diameter of the needles gradually changes from 100 to 250 nm (Figure 3A–IV). Interestingly, parallel needles in the RPE cell body are less organized than is seen in mammals (Figure 3A–III), and the distance between them varies (100–400 nm). The needle material is unknown and is more electron dense then melanin in birds. While some claim that the material is guanine, the guanine sandwich in fish crystallites appears completely white (Figure 3B–II and –IV), appearing as low density by electron microscopy, while the material in bird needles appears black.

*Crystallites* (sets of plates) are well represented in fish, both in elasmobranchs and in many teleosts [4, 6, 53–55] (Figure 3B). As an example, we present the reflection from walleye eye (Figure 3B–I), whose tapetum development was studied previously [5]. Figure 3B also shows crystallites in anchovy (a teleost) tapetum, (Figure 3B–II and -IV), and shark tapetum (elasmobranch, Figure 3B–III). At low magnification (×500, longitudinal section, Figure 3B–II) apical crystallites in anchovy RPE apical processes form a kind of pyramid (usually with 30° gradient inclination) that is complementary in shape to the photoreceptor OS cone, forming a sawtooth structure (Figure 3B–II, longitudinal section). The crystallite material is most often guanine [3]. These crystallite pyramids separate nearby photoreceptors (Figure 3B–II). On a transverse section (Figure 3B–IV), an anchovy photoreceptor OS (in the center) is shown surrounded by five crystallites (transverse section), which separates it from other OSs. Each crystallite consists of a stack of plates (sandwich structure, with guanine appearing white, Figure 3B–II and -IV), and guanine plate stacks (50–250 nm wide) are interspersed with other material (usually about 100–200 nm wide), forming a typical Bragg-type mirror. The melanin granules are situated between the crystallites (Figure 3B–IV). It was found that melanin granules in fish tapetum move (as part of retinomotor events) during light-dark adaptations, regulating the light reflectance by 30% to 85% [53]. Similarly, crystallites in shark (Figure 3B–III) also form Bragg-type mirrors (Figure 3B–III) and have melanin granules in between.

The diversity in tapetum position and structure encourages questions about the evolution of tapetum in specific vertebrate classes. [1, 2, 56, 57]. Comparative paleontological study performed by Schwab et al [57] suggested that tapetum had evolved in the Devonian period 345 to 395 million years ago at least in sharks, sturgeon, and lobe-finned fish either independently of each other or due to a common ancestor. Therefore, appearance of tapetum coincides with an explosion in the evolution of many different types of marine life. According to the authors, the choroidal tapetum was the first type of tapetum to evolve in vertebrates, with retinal tapeta appearing independently, while all have surprisingly similar mechanisms of light reflection. This suggestion coincides with conclusions of [1, 2].

## 7 | GENETIC VARIANTS, ATROPHY OF THE TAPETUM, SEASONAL CHANGES, TOXICOLOGY AND OTHER FACTORS AFFECTING REFLECTANCE

While some vertebrate species have reflective tapetum, in many cases, its presence and reflectance depend on genetics. We previously mentioned that cats have differing spectral reflections from tapetum, depending on their lineage [9, 10]. Also, some (white and Siamese) cats have reduced tapetum or no tapetum at all [35]. The same is well known in dogs: while the majority of dog strains have tapetum, it may be different in reflectance color in diverse strains and even within a single strain [58], and in some strains it is not present at all [59]. Moreover, diseases such as retinal atrophy in animals may lead to hyper-reflectance due to thinning of the retina on top of the tapetum [60]. By contrast, tapetal hypoplasia (a local disappearance of tapetum in certain spots) may lead to a reduction of eye reflection [61]. Interestingly, humans have no tapetum, and healthy humans have only low-level red reflectance from the choroid (visible in flash photos), but can reflect relatively well only in infra-red [62]. However, there are urban legends about humans (mainly children, see, eg, Gismodo) with shining reflective eyes. This is explicable, as patients with retinal cancer (retinoblastoma, usually pediatric) have visible reflection from cancerous cells in the retina [63]. Changes in eye reflection is also common if there are changes in retinal vasculature, as in Coats disease [64].

Interestingly, many (generally not toxic) substances can affect the tapetum and its reflectance. A macrolide antibiotic, rosamicin; an imidazo quinazoline (antibiotic); CGS 14796C (aromatase inhibitor) [65]; 1,3-di (4-imidazolino-2-methoxyphenoxy) propane lactate (antibiotic); and many other substances can dramatically reduce eye reflectance in dogs [66]. Histologically, these substances produce degeneration, thinning, or total loss of tapetal cells, with little or no alteration in pigment epithelium in general [66]. Spectrometry shows that even thermal stress may change optical properties of the tissue [67, 68].

In some animals, reflectance from the tapetum changes with the season. In arctic deer (Mammals), the color of reflectance from tapetum is greenish in summer, becoming deep blue in winter, and the change is explained as environmental adaptation and can be modeled by tapetum stretching in vitro [36].

## 8 | NANO-OPTICS OF THE VERTEBRATE TAPETUM

Throughout their scientific history, studies of the reflective properties of vertebrate eye mirrors have relied heavily on diffraction principles. If conventional optical components control light using gradual phase accumulation through propagation in refractive materials, diffracting devices rely on local phase shifts induced by subwavelength-scale optical components, in a field that is now termed “nano-optics.” It has been reported that the reflection of light by fish scales, as well as reflection by butterfly wings, chameleon skin or cuttlefish, uses the same diffraction-based principle [69–73]. Appreciation of the unconventionality of eye reflection came earlier. One hundred fifty years ago, it was shown that the cat eye tapetum produces reflection by “light interference.” In 1970, it was proposed that the two main principles of reflection in biological mirrors are: Mie-type reflection from wavelength-size spheres and Bragg-type multilayer subwavelength sandwiches [6]. Now there is a general understanding that diffractive optics prevail in biological reflectors, including different tapetum types [7, 8].

Recently, optical reflection from high-contrast gratings has been shown to be more effective than the reflection from conventional distributed Bragg reflectors, having a reflectivity of >99% over a broad wavelength range [74, 75]. A fully analytic solution for grating reflective properties was developed, focusing on this high reflectivity phenomenon [76]. It was shown that reflectivity is a function of light wavelength (W) as well as the periodic distance between rods (L), and the most effective reflectance occurs when the W/L ratio relation is in the range 2 to 2.5 [76], which corresponds well with what we have seen in carnivore and ungulate tapetums. Interestingly, practically no reflection occurs if the W/L ratio is >2.7. This finding suggests that dense gratings, such as collagen gratings in corneal cells, which have closely packed collagen fibrils with interfibrillary distances of 30 to 50 nm [77, 78], will have zero reflectance, enhancing cell transparency. Interestingly, the transparent tooth reported in deep-water dragonfish also has a similar highly periodic nanostructure [79]. This may be one of the principles that complements the classical view of transparency [45, 80].

## 9 | CONCLUSION

As a product of millions of years of evolution, the optics of vertebrate eyes is one of the wonders of science. The vertebrate eye was the inspiration for the development of conventional optics, and it may now stimulate the development of diffraction type-optical devices as well. On the other hand, the modern field of nanooptics can inform studies on the biology of eye optics, as many eye-related optical phenomenon remain unexplained. It is now clear that multilayer subwavelength gratings or sandwiches with periodic structures (photonic crystals) that shape light reflection in the *tapetum lucidum* of taxonomically diverse vertebrate animals are similar in principle to modern optical devices. This allows data on the microstructure of the tapetum to be used to improve our understanding of the visual system in general.

## Supplementary Material

fS1

fS2

fS3

## Data Availability

Data available on request from the authors
