# Supplementary material for: Multilayer subwavelength gratings or sandwiches with periodic structure shape light reflection in the tapetum lucidum of taxonomically diverse vertebrate animals
Source: J Biophotonics. Author manuscript; Available in PMC 2022 Sep 20. (PMC9487202; doi:10.1002/jbio.202200002)
Supplement: fS1 [file NIHMS1794294-supplement-fS1.pptx]

## Slide 1
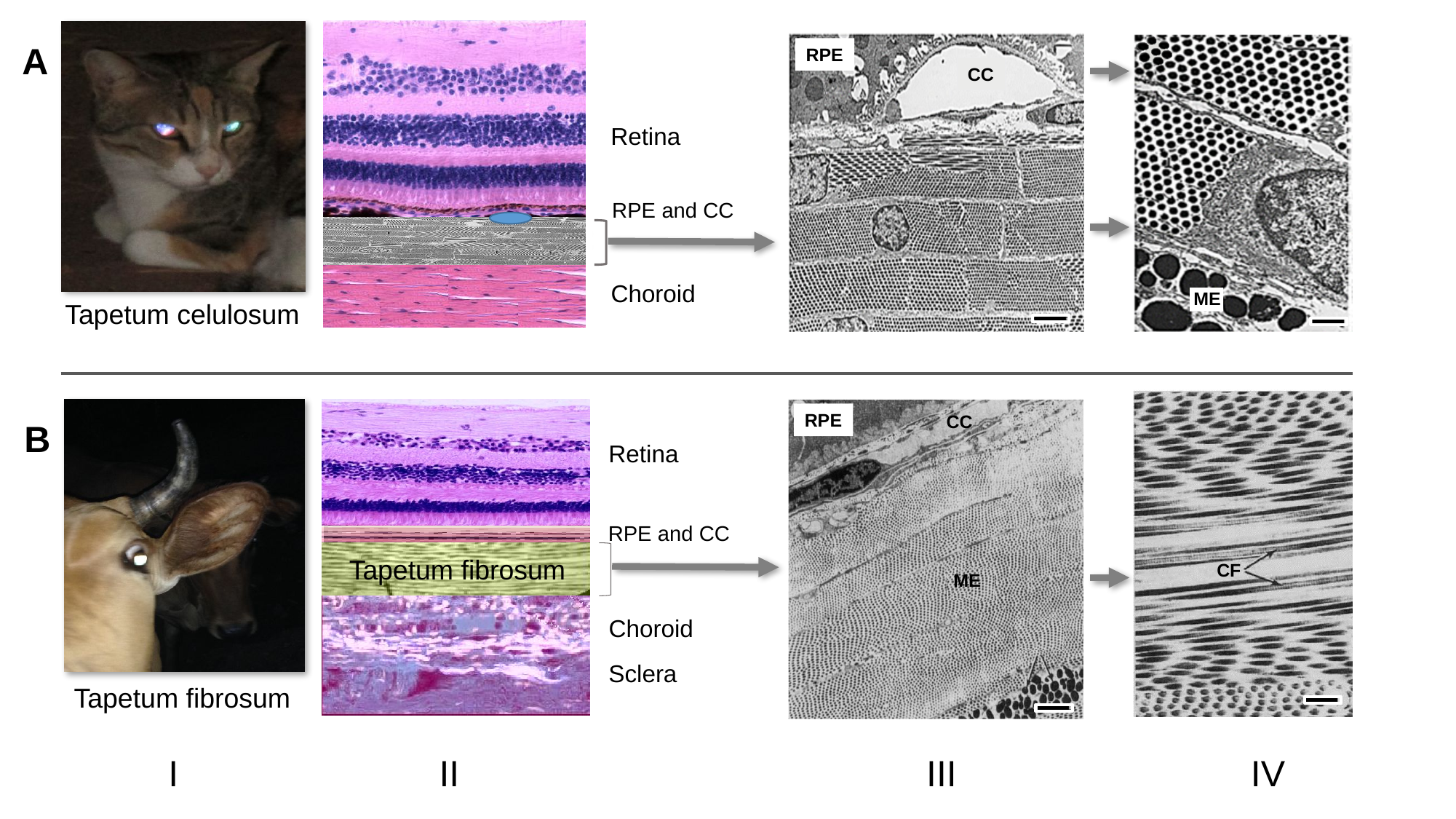

RPE
CC
ME
A
Retina
RPE and CC
Tapetum celulosum
Choroid
Tapetum celulosum
CF
Tapetum fibrosum
RPE
CC
ME
B
Retina
RPE and CC
Choroid
Sclera
Tapetum fibrosum
I
II
III
IV
