# Supplementary figures and images for: Multilayer subwavelength gratings or sandwiches with periodic structure shape light reflection in the tapetum lucidum of taxonomically diverse vertebrate animals
Source: J Biophotonics. Author manuscript; Available in PMC 2022 Sep 20. (PMC9487202; doi:10.1002/jbio.202200002)

## Slide 1
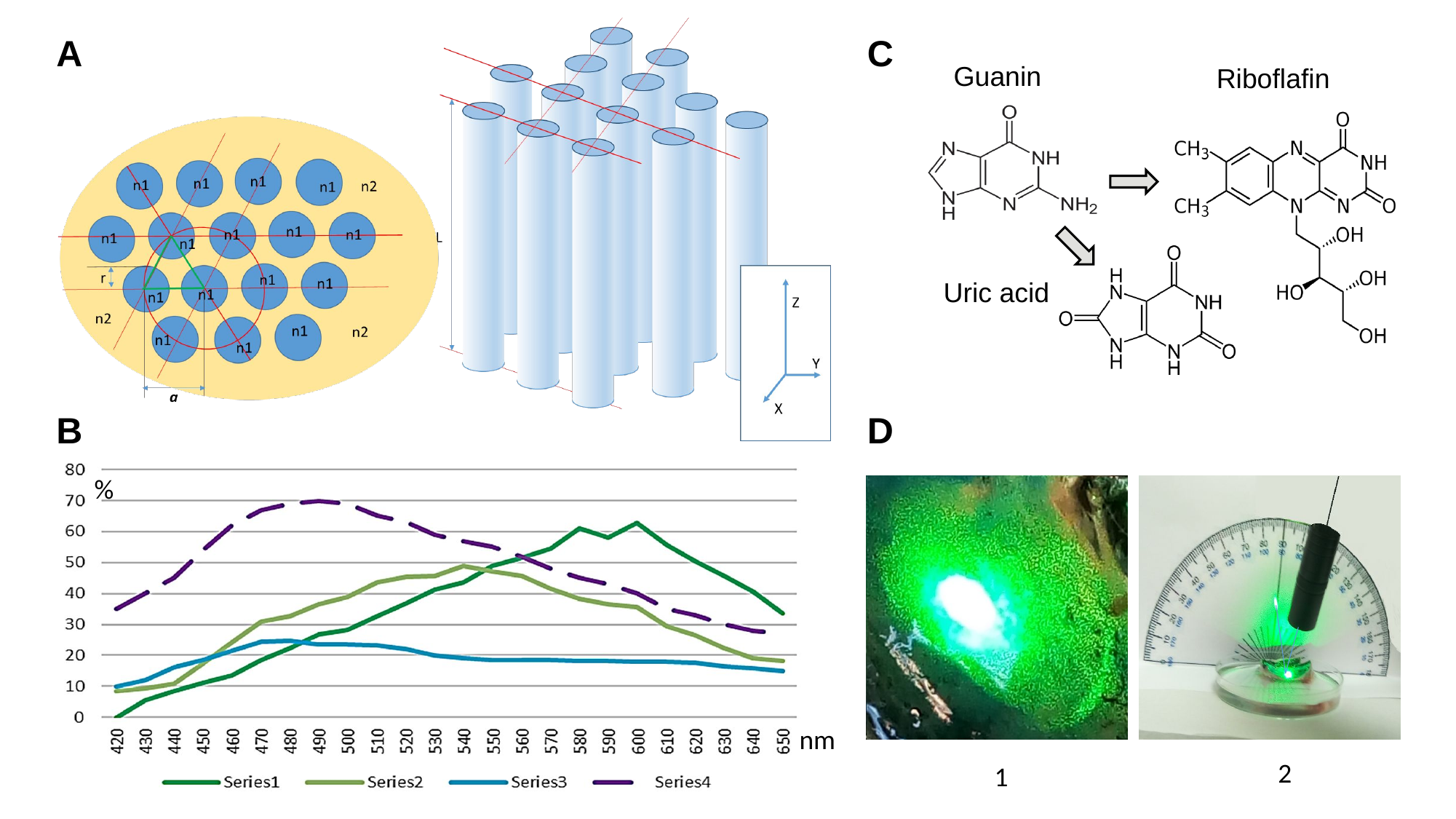

A
C
Guanin
Riboflafin
Uric acid
B
D
%
nm
2
1

Supplement: fS2 [file NIHMS1794294-supplement-fS2.pptx]
