# Supplementary material for: Multilayer subwavelength gratings or sandwiches with periodic structure shape light reflection in the tapetum lucidum of taxonomically diverse vertebrate animals
Source: J Biophotonics. Author manuscript; Available in PMC 2022 Sep 20. (PMC9487202; doi:10.1002/jbio.202200002)
Supplement: fS3 [file NIHMS1794294-supplement-fS3.pptx]

## Slide 1
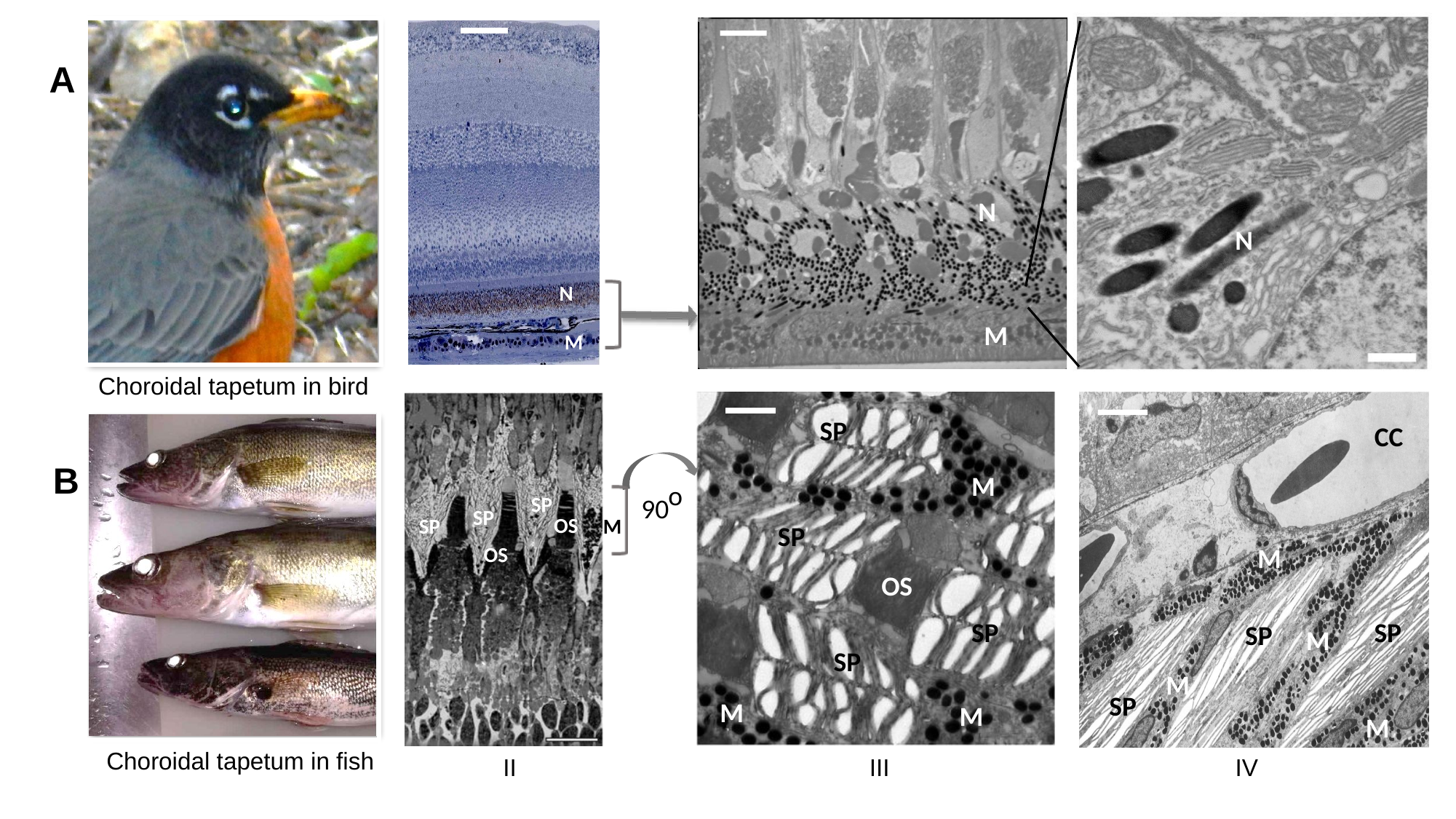

A
N
N
N
M
M
Choroidal tapetum in bird
SP
SP
OS
M
SP
OS
SP
CC
B
M
o
90
SP
M
OS
SP
SP
SP
M
SP
M
SP
M
M
M
Choroidal tapetum in fish
II
III
IV
